# Serious and Non-serious Hepatitis Reports in the U.S. Vaccine Adverse Event Reporting System, 1990–2026: A Cross-Sectional Analysis

**DOI:** 10.7759/cureus.117224

**Published:** 2026-09-30

**Authors:** Paula Aragón Gutierrez, Deepti Botlaguduru, Asritha Chowdary Medarametla, Sai Pratyusha Killy

**Affiliations:** 1 Dr. William C. Gorgas Faculty of Health Sciences, Universidad Latina de Panamá, Panama City, PAN; 2 Department of Population and Public Health Sciences, Keck School of Medicine, University of Southern California, Los Angeles, USA; 3 Internal Medicine, Kamineni Academy of Medical Sciences and Research Centre, Hyderabad, IND; 4 Obstetrics and Gynaecology, Southend University Hospital, Essex, GBR

**Keywords:** covid-19 vaccines, hepatitis, hepatitis b vaccine, passive surveillance, pharmacovigilance, post-marketing surveillance, serious adverse events, vaccine adverse events, vaers

## Abstract

Background

Post-marketing surveillance monitors reports of adverse events following vaccination, including reports of hepatitis. The Vaccine Adverse Event Reporting System (VAERS) is a passive surveillance system that can describe reporting patterns but cannot establish incidence, risk, or causality. This study aimed to characterize hepatitis reports received by VAERS based upon demographic characteristics, vaccine types, onset intervals, reported outcomes, seriousness classification, and temporal patterns in reporting.

Methodology

Data for this study were collected via a retrospective, cross-sectional analysis utilizing the Centers for Disease Control and Prevention’s Wide-ranging Online Data for Epidemiologic Research VAERS database. Reports of “Hepatitis” as the Medical Dictionary for Regulatory Activities (MedDRA) Preferred Term (code 10019717) were extracted from data that had been processed through April 24, 2026. Data for 2026 were partial because the database had been processed only through April 24, 2026. For reports where the reporting year could be determined, the time frame was 1990 to 2026. Data were categorized into frequency distributions and percentages for categorical variables. Pearson’s chi-square tests were conducted to compare serious vs. non-serious reports by age group, sex reported, and onset interval. Cramér’s V was calculated to quantify the magnitude of the differences between the groups. Due to the lack of mutual exclusivity among the categories for vaccine types and therefore the inability to treat them as independent data points, summary statistics describing the results for vaccine type were generated.

Results

A total of 529 hepatitis reports were retrieved. Of these 529 reports, 200 (37.81%) were designated as serious reports, while 329 (62.19%) were designated as non-serious reports. Individuals between the ages of 18 and 49 years comprised the greatest number of reports (n = 216; 40.83%). The female sex was identified in 261 (49.34%) reports. In 178 (33.65%) reports, the onset interval was unknown. Among those reports that documented an onset interval, one to three days was the most frequently documented onset interval (n = 70; 13.23% of all reports). Hepatitis B vaccines (n = 267; 50.47%) and COVID-19 vaccines (n = 134; 25.33%) were the two vaccine types most frequently documented. The seriousness classification was significantly different among the various age categories (χ²(5) = 21.61, p = 0.0006; Cramér’s V = 0.224). Neither sex (χ²(1) = 3.84, p = 0.0502; Cramér’s V = 0.089) nor onset interval (χ²(7) = 8.88, p = 0.2614; Cramér’s V = 0.159) were significantly related to seriousness classification. Vaccine-type findings were limited to descriptive comparisons because the categories were not mutually exclusive.

Conclusions

Reports of hepatitis that have been sent to the VAERS demonstrate differing classifications of seriousness based upon the age group. The vaccine-type findings were presented as descriptive summaries, as each report may contain multiple vaccines. Results presented here represent only patterns found in reports received by the VAERS; they do not provide an estimate of vaccine-specific risk, incidence, or causation. Further investigation into these findings will require additional research using denominator-based and clinically validated data.

## Introduction

In this study, hepatitis refers to a Vaccine Adverse Event Reporting System (VAERS) report coded with the Medical Dictionary for Regulatory Activities (MedDRA) Preferred Term “Hepatitis,” rather than a clinically confirmed diagnosis or an established vaccine-related adverse reaction. Given the widespread administration of vaccines, post-licensure surveillance is important for detecting and evaluating reported adverse events, including hepatic events [1]. Post-marketing surveillance data show sex-based disparities in spontaneous adverse reaction reports, with women reporting adverse reactions at a higher rate than men [2].

Describing the demographic distribution and reporting patterns of hepatitis reports following vaccination may help identify questions for further investigation. The mechanisms underlying hepatitis reported following vaccination remain uncertain, although immune-mediated processes similar to those involved in autoimmune hepatitis have been proposed [3-5]. Cases of liver damage have been documented after receiving several types of COVID-19 vaccines, and case reports and series have documented immune-mediated, autoimmune-like hepatitis [4]. A published case report described CD8 T-cell-dominant hepatitis occurring after SARS-CoV-2 vaccination [5]. In a multi-country case series, most patients had favorable outcomes; however, one patient developed fulminant liver failure requiring liver transplantation [4]. Most of the current research is conducted using case reports and small studies where people are followed for a short amount of time, and stratified analyses based on age, sex, type of vaccine, interval from vaccination to onset of symptoms, and seriousness of the report are rare, thus leaving a gap in real-world pharmacovigilance reporting data.

Rare or unexpected adverse events at scale need to be detected using big surveillance databases. VAERS, which is a national system for reporting adverse events by the public and healthcare providers, collects data on adverse events related to vaccines from different groups of people, different types of vaccines, and different time frames regardless of whether the reporter believes the vaccine caused the event [1]. A prior VAERS-based analysis examined reports of autoimmune hepatitis following COVID-19 vaccination [6]. The present study addresses a different question by describing reports coded with the MedDRA Preferred Term “Hepatitis” across vaccine types from 1990 through 2026 and comparing reporting patterns between serious and non-serious reports.

Therefore, the purpose of this study is to analyze hepatitis reports submitted to VAERS from 1990 through 2026. Specifically, this study aimed to evaluate demographic characteristics of hepatitis reports, reported clinical outcomes of hepatitis reports, vaccine types listed in hepatitis reports, and time intervals between vaccination and onset of symptoms. Additionally, this study aimed to compare reporting patterns between reports that were serious and reports that were not serious.

## Materials and methods

The present study is a retrospective, cross-sectional, pharmacovigilance analysis of VAERS reports. VAERS is a passive surveillance system maintained jointly by the Centers for Disease Control and Prevention (CDC) and the Food and Drug Administration (FDA) [1,7]. VAERS receives reports of adverse events associated with U.S.-licensed vaccines. Individuals who may submit reports include healthcare professionals, manufacturers of vaccines, patients, and members of the general public. Data for the current study were retrieved through the publicly available CDC Wide-ranging Online Data for Epidemiologic Research (WONDER) VAERS interface on May 10, 2026. At the time of retrieval, VAERS processed reports through April 24, 2026. The unit of analysis was a VAERS report containing the selected symptom term, rather than an individual patient or a clinically confirmed hepatitis case. As the data are publicly available in de-identified format and contain no personal identifiers, the current study was classified as non-human participant research according to 45 CFR 46 and Office for Human Research Protections guidance [8-10]. Therefore, institutional ethics committee approval was not required.

The CDC WONDER VAERS database was searched using the MedDRA Preferred Term “Hepatitis” (code 10019717) through the Data Search symptom selection interface. Among those reports with a known reporting year, the study period extended from 1990 through 2026. Data for 2026 were incomplete as the database had only been processed through April 24, 2026. Reports that lacked a known reporting year were included in the overall analysis; however, they were excluded from the analysis of temporal reporting patterns. The geographical area selected for inclusion in the present study was the United States and its territories, as well as reports where state/territory was unknown. This geographic scope was intentionally selected in the CDC WONDER VAERS query and was not an automatic restriction imposed by the database. There were no limits placed upon age, sex, or vaccine type. The primary endpoint was VAERS reports containing the MedDRA Preferred Term “Hepatitis” following a reported vaccination. The CDC WONDER query interface was utilized to create aggregate counts. According to CDC WONDER documentation, reports identified as duplicates or fraudulent are removed during routine VAERS processing [7]. Because this study used aggregate results from CDC WONDER, we did not independently verify that process, perform additional report-level duplicate detection, or assess whether secondary submissions remained in the data.

The following variables were retrieved through the query interface: age at vaccination as reported in VAERS (categorized as <1 year, 1-5 years, 6-17 years, 18-49 years, 50-64 years, 65+ years or unknown); sex (coded as male, female, or missing); vaccine type (with the 10 most frequently reported vaccine types presented in descending order); onset interval (defined as the number of days between vaccine administration and reported symptom onset categorized as 0 days, 1-3 days, 4-7 days, 8-14 days, 15-30 days, 31-60 days, 61-120 days, >120 days or unknown); event category (e.g., hospitalized, prolonged hospitalization, emergency room/office visit, permanent disability, life-threatening or death); and report seriousness (categorized as serious or non-serious). Seriousness was obtained from the CDC WONDER “Serious” variable, which classifies reports based on outcomes including death, permanent disability, life-threatening events, and hospitalization; the criteria also include prolonged hospitalization and, since June 30, 2017, congenital anomaly or birth defect [7]. Reports with missing or unknown values were maintained in descriptive summaries as distinct categories. Onset interval was obtained at the report level from CDC WONDER; we did not assign a separate interval to each vaccine product or dose listed in a report. As vaccine categories were not mutually exclusive (i.e., a single VAERS report may contain multiple vaccine products), vaccine-type counts represent vaccine listings rather than unique reports.

Statistical analyses were completed utilizing SAS OnDemand for Academics (SAS Institute Inc., Cary, NC, USA) [11]. Frequencies and percentages were used to summarize categorical variables. Comparisons were made between serious and non-serious reports across age group, reported sex, and onset interval. Pearson’s chi-square tests were employed for age group, reported sex, and onset interval. For each of these tests, observations represent individual reports, all expected cell counts were ≥5, and the expected frequency assumption was met. Vaccine-type findings were summarized descriptively because the categories were not mutually exclusive and vaccine listings could not be treated as independent observations.

Missing or unknown observations were retained in descriptive summaries; however, they were excluded from corresponding inferential analyses. The descriptive vaccine-type summary was restricted to the 10 categories. All tests were two-tailed, and statistical significance was defined as p-value <0.05. Degrees of freedom were reported for Pearson’s chi-square tests. Cramér’s V was computed from Pearson’s chi-square statistic as a measure of the strength of association between categorical variables. A 95% confidence interval (CI) for Cramér’s V in the sex-by-seriousness comparison was estimated using the noncentral chi-square method.

## Results

A total of 529 hepatitis reports were submitted to VAERS. Among reports with a known reporting year, the years ranged from 1990 to 2026. Data for 2026 were incomplete because the database had been processed through April 24, 2026. The reporting year was unknown for 109 (20.60%) reports. Of the 529 reports, 200 (37.81%) were classified as serious and 329 (62.19%) as non-serious. The analyses included demographic characteristics, reported outcomes, intervals between vaccination and symptom onset, listed vaccine types, and temporal reporting patterns.

Table 1 shows the age distribution of individuals described in the reports. The largest proportion involved individuals aged 18-49 years (n = 216; 40.83%), followed by reports with unknown age (n = 99; 18.71%) and those involving individuals aged 50-64 years (n = 75; 14.18%). Reports involving children younger than one year represented the smallest age group (n = 15; 2.84%). Sex distribution is also shown in Table 1. Female sex was recorded in 261 (49.34%) reports, male sex in 219 (41.40%), and sex was missing in 49 (9.26%).

**Table 1:** Demographic characteristics of hepatitis reports submitted to VAERS. VAERS = Vaccine Adverse Event Reporting System

Variable	Value n (%)
Total reports	529
Age	
<1 year	15 (2.84)
1–5 years	33 (6.24)
6–17 years	49 (9.26)
18–49 years	216 (40.83)
50–64 years	75 (14.18)
65+ years	42 (7.94)
Age unknown	99 (18.71)
Sex	
Male	219 (41.40)
Female	261 (49.34)
Missing	49 (9.26)

The annual distribution of the 420 reports with a known reporting year is shown in Figure 1. The highest number of submitted reports occurred in 2021 (68 reports; 12.85% of all 529 reports), followed by 2022 (34 reports; 6.43%). One report was recorded for 2025 and one for 2026 (0.19% of all reports each). Data for 2026 were incomplete because the database had been processed only through April 24, 2026. Reporting and processing delays may further limit the 2026 count, which should not be compared directly with counts for complete years. Reports with unknown reporting years (n = 109) were excluded from the figure.

**Figure 1:**
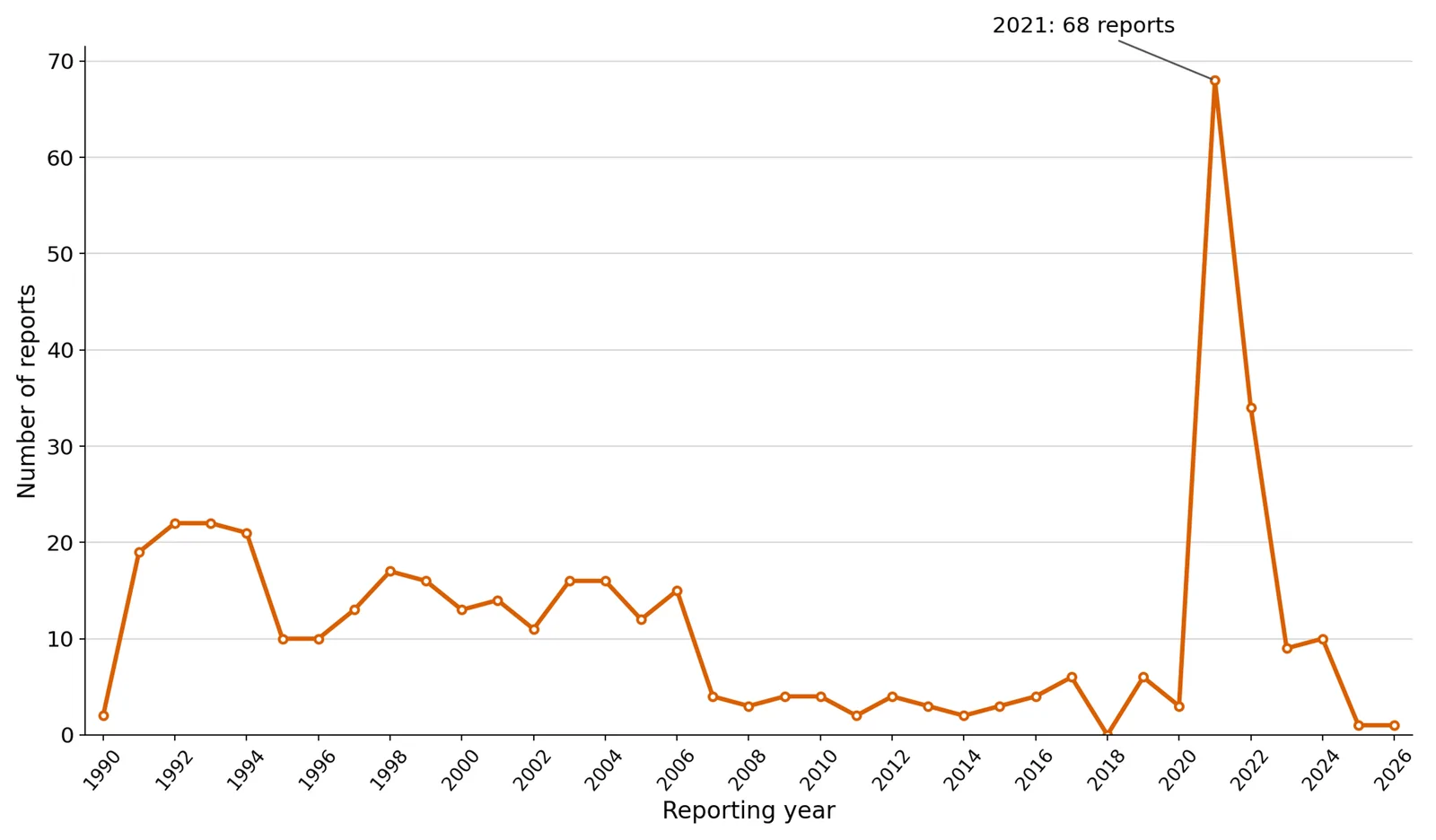
Annual hepatitis reports submitted to VAERS. Annual number of hepatitis reports submitted to VAERS by reporting year. Reports with an unknown reporting year (n = 109) were excluded from this figure. Data for 2026 were partial because the database had been processed through April 24, 2026. VAERS = Vaccine Adverse Event Reporting System

The most frequently listed vaccine types among the hepatitis reports are shown in Table 2. Hepatitis B vaccines were listed in the largest proportion of reports (n = 267; 50.47%), followed by COVID-19 vaccines (n = 134; 25.33%) and hepatitis A vaccines (n = 34; 6.43%). The “COVID-19 vaccine” category was used as provided by CDC WONDER; the authors did not group individual products.

**Table 2:** Top 10 vaccine types listed in VAERS hepatitis reports, ranked by frequency, n (%). Percentages were calculated using all 529 hepatitis reports as the denominator. Only the 10 most frequently listed vaccine types are presented. Vaccine categories were not mutually exclusive; counts represent vaccine listings rather than unique reports. VAERS = Vaccine Adverse Event Reporting System

Rank	Vaccine type	n (%)
1	Hepatitis B vaccine	267 (50.47)
2	COVID-19 vaccine	134 (25.33)
3	Hepatitis A vaccine	34 (6.43)
4	Measles, mumps, and rubella virus vaccine, live	21 (3.97)
5	Varivax-varicella virus live	19 (3.59)
6	Influenza virus vaccine, trivalent (injected)	15 (2.84)
7	Hepatitis A and hepatitis B vaccine	10 (1.89)
8	Influenza virus vaccine (no brand name)	10 (1.89)
9	Haemophilus B conjugate vaccine	9 (1.70)
10	Pneumococcal vaccine, polyvalent	8 (1.51)

The onset interval of 178 (33.65%) reports was not reported. The most common reported onset interval was one to three days after vaccination (n = 70; 13.23%), followed by onset on the same day as vaccination (n = 49; 9.26%), and four to seven days after vaccination (n = 47; 8.88%). Overall, 44 (8.32%) reports reported an onset interval of greater than 120 days. Outcome categories were not mutually exclusive. The category “None of the above” was recorded in 184 (34.78%) reports, whereas 164 (31.00%) reports included an emergency room or office visit and 154 (29.11%) included hospitalization. Other reported outcomes included life-threatening events (n = 53; 10.02%), permanent disability (n = 29; 5.48%), and death (n = 26; 4.91%). Overall, 200 (37.81%) reports were considered serious, and 329 (62.19%) were considered non-serious. These findings are summarized in Table 3.

**Table 3:** Reported onset intervals, outcome categories, and seriousness classifications among VAERS hepatitis reports. Outcome categories were not mutually exclusive; therefore, their counts and percentages may sum to more than 529 and 100%, respectively. Percentages were calculated using all 529 reports as the denominator. VAERS = Vaccine Adverse Event Reporting System

Onset interval	n (%)
0 days	49 (9.26)
1–3 days	70 (13.23)
4–7 days	47 (8.88)
8–14 days	39 (7.37)
15–30 days	45 (8.51)
31–60 days	27 (5.10)
61–120 days	30 (5.67)
Over 120 days	44 (8.32)
Unknown	178 (33.65)
Event category	
None of the above	184 (34.78)
Emergency room/Office visit	164 (31.00)
Hospitalized	154 (29.11)
Life-threatening	53 (10.02)
Permanent disability	29 (5.48)
Death	26 (4.91)
Existing hospitalization prolonged	9 (1.70)
Serious	
Yes	200 (37.81)
No	329 (62.19)

Age group was significantly associated with whether reports were classified as serious or non-serious (χ²(5) = 21.61, p = 0.0006; Cramér’s V = 0.224). Within each age group, 10 of 15 (66.7%) reports among children younger than one year, 22 of 33 (66.7%) among children aged 1-5 years, 27 of 49 (55.1%) among children aged 6-17 years, 79 of 216 (36.6%) among adults aged 18-49 years, 24 of 75 (32.0%) among adults aged 50-64 years, and 20 of 42 (47.6%) among adults aged 65 years or older were classified as serious. Reported sex was not statistically significantly associated with seriousness classification at the predefined α = 0.05 level (χ²(1) = 3.84, p = 0.0502; Cramér’s V = 0.089, 95% CI: 0.000-0.179). No statistically significant difference was detected in the distribution of reported onset intervals between serious and non-serious reports (χ²(7) = 8.88, p = 0.2614; Cramér’s V = 0.159). The distribution of serious and non-serious vaccine listings across the 10 vaccine types is presented descriptively in Table 4. COVID-19 vaccines accounted for 68 serious listings, followed by hepatitis B vaccines with 67 and hepatitis A vaccines with 15.

**Table 4:** Comparison of serious and non-serious hepatitis reports by demographic characteristics, onset interval, and vaccine type. χ² = Pearson’s chi-square statistic; parentheses indicate degrees of freedom; V = Cramér’s V. *: p < 0.05 indicates statistical significance. Reports with unknown values were excluded from the corresponding statistical comparison. Vaccine categories were not mutually exclusive because a single VAERS report could include more than one vaccine product. Therefore, vaccine-type counts represent vaccine listings and may exceed the number of unique reports. VAERS = Vaccine Adverse Event Reporting System

Variable	Serious	Non-serious	Test statistic and effect size	P-value
Age				
<1 year	10	5	χ²(5) = 21.61; V = 0.224	0.0006^*^
1–5 years	22	11		
6–17 years	27	22		
18–49 years	79	137		
50–64 years	24	51		
65+ years	20	22		
Sex				
Male	99	120	χ²(1) = 3.84; V = 0.089	0.0502
Female	95	166		
Vaccine type				
Hepatitis B vaccine	67	200	Descriptive analysis only	N/A
COVID-19 vaccine	68	66		
Hepatitis A vaccine	15	19		
Measles, mumps, and rubella virus vaccine, live	14	7		
Varivax-varicella virus live	15	4		
Influenza virus vaccine, trivalent (injected)	9	6		
Hepatitis A and hepatitis B vaccine	3	7		
Influenza virus vaccine (no brand name)	5	5		
Haemophilus B conjugate vaccine	8	1		
Pneumococcal vaccine, polyvalent	2	6		
Onset interval				
0 days	16	33	χ²(7) = 8.88; V = 0.159	0.2614
1–3 days	31	39		
4–7 days	22	25		
8–14 days	21	18		
15–30 days	26	19		
31–60 days	13	14		
61–120 days	17	13		
Over 120 days	18	26		

## Discussion

The current study examined reports of hepatitis submitted to VAERS between 1990 and 2026. The study evaluated demographic characteristics, reported outcomes, onset intervals, vaccine-type distribution, temporal reporting patterns, and differences between reports classified as serious and non-serious. A total of 529 hepatitis reports were found. Reports involving adults aged 18-49 years and women were the largest demographic groups, while reports in the pediatric age group had higher percentages of reports classified as serious. The hepatitis B vaccine had the largest number of vaccine listings.

Overall, 37.81% of reports were classified as serious. Reported outcomes included hospitalization, life-threatening events, permanent disability, and death. The largest number of reports with a known reporting year occurred in 2021, during the period of widespread COVID-19 vaccination. Age group was significantly related to seriousness classification, while reported sex and onset interval were not. In addition, the distribution of serious and non-serious vaccine listings was summarized descriptively across vaccine types. Reports involving adults aged 18-49 years made up the largest age group and accounted for 40.83% of all hepatitis reports (Table 1). However, as VAERS does not collect information on the number of vaccine doses administered within each demographic group, the observed distribution cannot be interpreted as evidence of increased incidence or risk in working-age adults. This pattern may reflect differences in vaccination exposure, population size, reporting behavior, or other factors. Active surveillance systems such as the Vaccine Safety Datalink may help address this limitation [12].

Women accounted for 49.34% of reports, while men accounted for 41.40%, and sex was missing in 9.26% (Table 1). A greater percentage of reports among women has also been noted in U.S. COVID-19 vaccine safety monitoring data [13]. One hypothesis for observed differences in outcomes between sexes is that there may be differences in their respective immune response to vaccines and/or their ability to report adverse events. However, the data used in the present study were aggregate data and therefore could not provide insight into either of these possibilities [2,14]. There was no statistically significant relationship found between the reported sex of an individual who experienced an adverse event after receiving a vaccine and whether or not the classification of the seriousness of the event was “serious” (p = 0.0502; see Table 4). The proportion of reported serious adverse events was higher among males compared to females. As stated previously, this result did not reach the predetermined level of statistical significance. Thus, this finding cannot be taken as proof of a difference in risk based on sex. Among all 529 hepatitis reports, hepatitis B vaccines were listed in 267 (50.47%) reports, followed by COVID-19 vaccines in 134 (25.33%) reports (Table 2). One possible explanation for the high number of hepatitis B vaccine listings is its longer history of use and administration across different age groups [15]. VAERS does not collect information regarding how many people receive each vaccine; therefore, it is impossible to compare reporting rates or specific risks associated with individual vaccines [7].

Among the vaccine listings shown in Table 4, COVID-19 vaccines accounted for 68 serious and 66 non-serious listings, while hepatitis B vaccines accounted for 67 serious and 200 non-serious listings. These findings were summarized descriptively, and no inferential comparison was performed across vaccine types. Vaccine categories were not mutually exclusive, and the independence of vaccine-related data points could not be established. The results only provide insight into how frequently certain vaccines are reported to VAERS and do not suggest that any one type of vaccine presents a higher risk for developing hepatitis or other serious liver problems. Several reports have been published regarding the development of autoimmune hepatitis-like conditions following COVID-19 vaccinations. These reports present possible mechanisms including molecular mimicry, bystander immune activation, and inflammatory responses [16,17]. The data used in the study were aggregated data and therefore could not confirm these diagnoses or assess the proposed mechanisms.

Age group was significantly associated with seriousness classification (χ²(5) = 21.61; p = 0.0006; Cramér’s V = 0.224) (Table 4). In this dataset, the Cramér’s V value suggests a modest association between age group and seriousness classification. Of the 15 reports for children less than one year old, 10 (66.7%) were classified as serious. Similarly, 22 of 33 (66.7%) reports for children aged 1-5 years and 27 of 49 (55.1%) reports for children aged 6-17 years were classified as serious. In contrast, 79 of 216 (36.6%) reports for adults aged 18-49 years were classified as serious. Acute liver failure in children can result in severe clinical outcomes [18], and differences in organ function and drug metabolism in children are well known [19]. However, these factors do not explain the reporting pattern seen in this study. The absence of age-specific vaccination denominators, the relatively small number of pediatric reports, and possible differences in reporting behavior prevent drawing conclusions about age-specific incidence or susceptibility.

Onset intervals were spread across all measured post-vaccination time frames (Table 3). Onset interval was unknown in 178 (33.65%) reports. Among all 529 reports, the most commonly recorded known interval was 1-3 days after vaccination (n = 70; 13.23%), followed by same-day onset (n = 49; 9.26%). Reports were also filed at 15-30 days (n = 45; 8.51%), 61-120 days (n = 30; 5.67%), and more than 120 days after vaccination (n = 44; 8.32%). Onset interval was not significantly associated with seriousness classification (χ²(7)= 8.88; p = 0.2614; Cramér’s V = 0.159) (Table 4). The analysis did not provide evidence of a difference in onset interval distribution between serious and non-serious reports. Although this study could not identify the biologically plausible risk period, there have been several case studies regarding autoimmune-like hepatitis following vaccination, and those studies indicated a wide range of time periods between receipt of the vaccine and development of symptoms [4,17]. Due to the absence of clinical evaluations of each VAERS report and the numerous reports that lacked information on the onset interval, we could not draw any conclusions regarding causal relationships or underlying mechanisms.

Serious reports comprised 37.81% of the dataset (Table 3). Hospitalization was reported in 154 (29.11%) reports, life-threatening events in 53 (10.02%), permanent disability in 29 (5.48%), and death in 26 (4.91%). As VAERS outcome categories are not mutually exclusive, a single report may be included in multiple categories. Additionally, an outcome listed in a VAERS report does not mean that vaccination caused or contributed to that outcome. Immune-mediated adverse reactions to vaccines have been described in the published literature [20]. Reports specifically concerning COVID-19 vaccination describe a broad clinical spectrum of immune-mediated liver injury, ranging from elevated hepatic enzyme levels to severe hepatic dysfunction [4,21]. The aggregate VAERS data used in this study did not allow for verification of diagnoses, evaluation of baseline liver disease, or determination of whether reported outcomes were attributable to vaccination.

Temporal analysis demonstrated an increase in reports of hepatitis in 2021 with a total of 68 reports (Figure 1). Although there was an increase in reports of hepatitis during the period of large-scale COVID-19 vaccine administration, this does not mean that the administration of COVID-19 vaccines caused an increase in reports of hepatitis. Additionally, the sheer volume of COVID-19 vaccines given to the public and increased safety monitoring associated with the mass vaccination effort may have altered the reporting process for hepatitis [22]. Public awareness and media coverage of hepatitis could increase the number of reports received by passive surveillance systems [7]. The Weber effect, characterized by increased spontaneous reporting following the introduction of a new medical product, may also have influenced reporting patterns [23]. As VAERS has no unvaccinated control group and no information regarding background hepatitis incidence or the number of vaccine doses administered, this study is unable to differentiate whether the observed increase in serious adverse event reports represents a true safety signal or if it represents changes in either vaccine exposure or reporting behaviors.

Comparisons between serious and non-serious reports showed a significant relationship between age group and seriousness classification (p = 0.0006). The distribution of serious and non-serious vaccine listings across vaccine types was evaluated descriptively. Reported sex was not significantly associated with seriousness (p = 0.0502), and onset interval also failed to show a significant association (p = 0.2614) (Table 4). These results demonstrate the value of using stratified pharmacovigilance analyses to characterize reporting patterns and develop hypotheses. However, they should not be used to identify causal risk factors or determine which vaccine products are more likely to cause hepatitis or serious outcomes. Future studies utilizing clinically adjudicated hepatitis definitions, vaccine administration denominators, appropriate vaccinated and unvaccinated populations or other comparator groups, patient-level data, and active surveillance systems such as the Vaccine Safety Datalink are needed to address these issues [12].

Limitations

This study has several limitations. As a passive surveillance system, VAERS is subject to underreporting, incomplete reporting, stimulated reporting, and reporting bias. Filing a report with VAERS does not mean that a vaccine caused or contributed to hepatitis or any reported outcome [1,7]. Because the data did not provide denominators representing the number of vaccine doses administered, incidence rates and vaccine-specific risks could not be calculated or compared.

Onset interval was unknown in 33.65% of reports, age was unknown in 18.71%, and sex was missing in 9.26%, limiting the precision of the temporal and demographic analyses. Reports with missing or unknown values were omitted from the corresponding statistical comparisons. Consequently, the comparisons for onset interval, age, and sex were based on different subsets of reports. If reports with missing values differed from those with available data, these subsets may not fully represent all 529 reports. Excluding reports also reduced the sample size available for each comparison, which may have limited statistical power, particularly for onset interval, where missing data were most frequent. Because individual VAERS reports could include more than one vaccine product, vaccine categories were not mutually exclusive and vaccine-level observations were not necessarily independent.

Vaccine type count represents vaccine listings and not individual reports. Therefore, vaccine-type findings were limited to descriptive summaries, and no inferential comparison was performed across vaccine types. Analyses were performed on aggregates from the VAERS query interface. Manual review could not be done by the authors for possible duplicate reports, clinical accuracy, diagnostic confirmation, and/or alternative causes of hepatitis. Although CDC WONDER states that duplicate events and false reports are deleted as part of the routine processing of VAERS data, the authors were unable to independently verify the deletion of duplicates and/or identify additional secondary reports.

The aggregate VAERS data did not provide information regarding comorbidities, prior liver disease, concomitant medication use, laboratory test results, histopathologic confirmation, disease progression, or treatment outcomes. Additionally, an increase in public awareness of the COVID-19 vaccine, an increase in media coverage of the COVID-19 vaccine, an increase in the number of vaccinations administered, and an increase in stimulated reporting of adverse events following vaccination during the COVID-19 pandemic may represent a shift in the pattern of reporting since 2020. A larger number of individuals vaccinated and reported over a longer time frame may also alter the ability to compare reporting trends across different time frames.

## Conclusions

The analysis identified 529 VAERS reports coded with the MedDRA Preferred Term “Hepatitis” from 1990 to 2026. Of those reports, 37.81% (200 reports) were classified as serious. The data collected for 2026 were incomplete due to processing of the database only up until April 24, 2026. Adults aged 18-49 years were the most frequently reported age group, and female sex was recorded more frequently than male sex among submitted reports. Pediatric age groups reported higher proportions of serious reports than did adults. Hepatitis B vaccine was the most frequently listed vaccine type among the vaccine listings in hepatitis reports. Age group was found to be statistically significantly related to seriousness classification. Sex and onset interval were not found to be statistically significantly related to seriousness classification. The distribution of serious and non-serious vaccine listings across vaccine types was summarized descriptively because the categories were not mutually exclusive. Reports were recorded across all onset interval categories. The largest number of reports with a known reporting year occurred in 2021, during the period of widespread COVID-19 vaccination. These results provide information regarding reporting patterns in VAERS but cannot be used to estimate incidence rates, vaccine-specific risks, or causal relationships. Additional studies utilizing vaccination denominators, appropriate comparative populations, patient-specific clinical data, laboratory test results, and histologic examination are required to determine possible etiologic factors and mechanisms of injury.
